# Simple and accessible methods for quantifying isolated mucins for further evaluation

**DOI:** 10.1016/j.mex.2025.103267

**Published:** 2025-03-22

**Authors:** Hannah J. McIntire-Ray, Elex S. Rose, Stefanie Krick, Jarrod W. Barnes

**Affiliations:** a Gregory Fleming James Cystic Fibrosis Research Center, Univ. of Alabama at Birmingham, Birmingham, AL, USA; b Department of Medicine, University of Alabama at Birmingham, Birmingham, AL, USA

**Keywords:** Isopycnic ultracentrifugation, Mucin quantitation, Agarose-polyacrylamide Gel electrophoresis, Ag-PAGE, Mucin biology, Salivary MUC5B, Isolation, Quantitation, and Evaluation of Salivary MUC5B

## Abstract

In this study, we present a detailed workflow for the isolation, quantitation, and evaluation of mucin proteins. These methods are applicable to a variety of biological, mucin-containing samples from the airways and other mucosal organ systems. While this report focuses on the salivary MUC5B protein from the respiratory system, the presented methodologies can be applied to other mucins, contributing to a broader application of these techniques. We used a simplified isopycnic centrifugation to purify and enrich MUC5B from human saliva. Isolated MUC5B was then subjected to a Bradford protein assay using a bovine submaxillary mucin (BSM) standard, which more accurately reflects the mucin concentration in our samples compared to a bovine serum albumin (BSA) standard. Additionally, we compare the mucin levels following quantitation using agarose polyacrylamide gel electrophoresis. Our findings show a near 2-fold increase in quantitation from the more representative, BSM standard, suggesting its importance for mucin studies. These methods support a wide range of experimental applications looking to assess mucins, thereby contributing to the broader field of mucin studies and advancing our understanding of the implications of mucins in health and disease.

• A streamlined, one-step isopycnic ultracentrifugation to isolate MUC5B from human saliva
• A Mucin Bradford assay that is modified from existing Bradford assay techniques to better quantitate mucin for mucin studies
• An agarose-polyacrylamide gel electrophoresis method used to visualize and confirm the isolation and quantitation of mucin

## Graphical abstract

**Figure:**
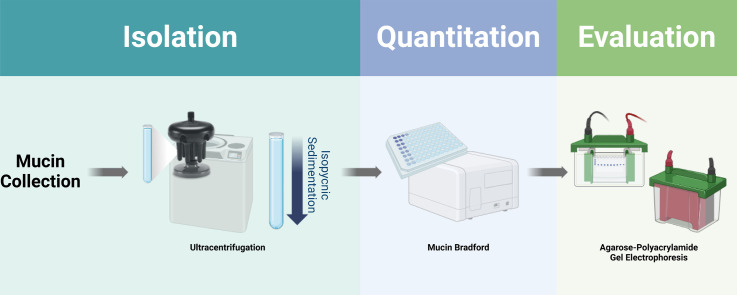

Specifications table

**Table:** 

Subject area:	Biochemistry, Genetics and Molecular Biology
More specific subject area:	Mucin Biology
Name of your method:	Isolation, Quantitation, and Evaluation of Salivary MUC5B
Name and reference of original method:	**Isopycnic Ultracentrifugation** Thornton DJ, Khan N, Mehrotra R, et al. Salivary mucin MG1 is comprised almost entirely of different glycosylated forms of the MUC5B gene product. Glycobiology. 1999;9(3):293–302. **Agarose-Polyacrylamide Gel Electrophoresis** Issa SM, Schulz BL, Packer NH, Karlsson NG. Analysis of mucosal mucins separated by SDS-urea agarose polyacrylamide composite gel electrophoresis. Electrophoresis. 2011 Dec;32(24):3554–63. doi: 10.1002/elps.201100374. Epub 2011 Nov 24. PMID: 22,120,911.
Resource availability:	Listed below within the corresponding methods.

## Background

Epithelial surfaces exposed to the external environment are protected by an extracellular hydrogel called mucus. Mucus lines the epithelium in several major organs including the airway, gastrointestinal tract, reproductive tract, and ocular membranes. Within these organs, mucus serves several roles to maintain epithelial health including lubrication, hydration, and as the first line of immunity against pathogens [[1], [2], [3], [4], [5]]. Although the function of mucus is to protect, several disease conditions lead to pathologic mucus, which often causes more damage than the primary disease itself [2,6,7]. In cystic fibrosis (CF), for example, a buildup of thick and adherent mucus leads to impaired mucociliary clearance thereby promoting bacterial colonization, infection, and lung damage that leads to mortality in those with CF. The main structural components of mucus that provide its rheological characteristics are large glycoproteins called mucins [1,2].

Although the exact components of mucus vary across tissues, mucins are the principal component that provide the structure of the mucus gel. Mucins are large glycoproteins (up to 50 MDa) with glycan carbohydrates contributing up to 80 % of their total mass [1,2,6]. Mucins and their extensive glycosylation greatly provide the rheological framework of mucus, and therefore, play a critical role in disease states plagued by viscous and adherent mucus [[8], [9], [10]]. Mucins are expressed by 21 known mucin genes that can broadly be divided into two families: 1) gel-forming mucins that are polymeric (with the exception of MUC7) and form the mucus gel layer and 2) tethered mucins that contain a transmembrane domain that keeps them anchored to the epithelial surface [1,2,11,12]. Aberrant mucin properties primarily pertaining to protein expression, glycosylation patterns, and macromolecular conformation contribute to the pathologic mucus that cause several diseases. Therefore, methods to accurately determine mucin levels are essential to propagate the discovery of mucin abnormalities in disease and to develop therapeutic strategies to better treat them.

Here, we present a purification and quantitation method that provides a more reliable and consistent assessment of mucin levels across various sources, experimental setups, and conditions. While these methods can be applied to mucins isolated from various biological samples including saliva, sputum, mucus, and tissue/cells from lung and other mucosal organ systems, these methods will isolate, quantitate, and evaluate salivary MUC5B protein. We provide a stepwise method for mucin purification using isopycnic centrifugation followed by mucin quantitation that is more accurate when using a similarly structured protein, like bovine submaxillary mucin (BSM), as a protein standard rather than the conventionally used bovine serum albumin (BSA). We find that differences in standard selection are notable where BSM as a standard for mucins will help to more accurately quantitate mucin for experiments that are limited and/or exact concentration/equivalent amounts are needed for analysis. In particular, rate-zonal centrifugation, mass spectrometry, along with other molecular assays often used in mucin studies [13] depend on a high degree of accuracy and consistency to achieve optimum results. To show this, isolated and quantified mucins were analyzed using agarose-polyacrylamide gel electrophoresis (Ag-PAGE), a common technique for mucin evaluation under different conditions [14,15], to visualize the difference in sample loading from the different quantitation methods. Together, these methods provide tools for a range of experimental applications for mucins that largely contribute to the accessibility and reproducibility of mucin studies in health and disease.

## Method details

### Isopycnic isolation of mucin from saliva

#### Sample collection and preparation

The source of mucus collection is critical to determine the types of mucins present and the specific processing steps required prior for isolation. For example, a collection of sputum will primarily contain MUC5B and MUC5AC, with lesser amounts of MUC7 and the tethered mucins MUC16, MUC4, MUC1, and MUC20. On the other hand, a collection of saliva will primarily contain MUC5B with smaller amounts of MUC7 and potentially trace amounts of MUC19, MUC1, and MUC4 [1,[16], [17], [18]]. Mucus collections containing a mixture of soluble and insoluble mucins, as mentioned, may require the addition of chaotropic and/or reducing agents [19,20]. Furthermore, depending on the outcome measures, mucins can be isolated in a native state or under denatured conditions. Native conditions may be preferred if tertiary/quaternary structure, protein interactions, and/or ionic interactions are being studied [15,21]. For this purpose, we natively isolated the soluble portion of salivary MUC5B, using a slightly modified protocol based on previous established methods [15,21,22]. In the case of saliva, as outlined in the methods below, IRB approval is needed and samples should be treated as biohazard. Please follow all institutional policies related to working with saliva and make special note if potentially infectious or contaminated with blood as this is considered biohazardous.

**Materials:**

• Parafilm (Sigma-Aldrich, #P7793).
  **Note:** Store parafilm away from other chemical reagents in the laboratory.
• 50 mL Conical Tube (ThermoFisher Scientific, #339,652)
• Tabletop Centrifuge (ThermoFisher Scientific, Sorvall ST 40R)
• Solubilization Buffer – pH 7.4
  • 150 mM Sodium Chloride (Fischer, #L-23,071)
  • 30 mM Tris–HCl (Sigma, #RDD008)
  • 1.5X Protease Inhibitor
    • EDTA-free Protease Inhibitor Cocktail (Millipore-Sigma, #11,836,170,001)
    • Protease Inhibitor Cocktail (Millipore-Sigma, #11,836,153,001)
• Tube Rotator (ThermoFisher Scientific, #88,881,001)

**Methods**:

1. Stimulate saliva secretion by chewing on parafilm and spit into a conical tube on ice until adequate sample is collected. A single prep following this protocol requires approximately 3.3 mL of saliva for one isopycnic gradient.
  **Note:** Do not swallow parafilm.
  **Note:** Do not use parafilm if you require unstimulated saliva secretions: however, the yield may be lower.
2. Clarify saliva by placing tube in a 4 °C centrifuge and spin at 3000x*g* for 30-minutes to sediment salivary debris. Pour off supernatant into a new tube.
  **Note:** Dispose of pellet per institution guidelines related to working with saliva.
3. Add Solubilization Buffer with protease inhibitors to clarified saliva.
  **Note:** A 2:1 dilution of Solubilization Buffer to saliva sample is found to be optimal for mucin solubilization without over-diluting the sample for further analysis. If less saliva sample is collected (<3.3 mL), bring volume up to roughly 10 mL to ensure enough volume of sample for the proceeding steps.
  **Note:** Protease inhibitors are recommended to prevent degradation of sample; however, depending on the study endpoint, EDTA may not be recommended. EDTA, due to its chelation properties, may alter the structural conformation of mucin from the well-defined relationship between mucin structure and cation interactions [15,23].
4. Place sample on rotator at 4 °C overnight.
5. Centrifuge in a pre-cooled 4 °C centrifuge at 4400x*g* for 30-minutes.
6. Save supernatant for use in gradient.

#### Isopycnic (Cesium chloride) centrifugation

Cesium chloride (CsCl) isopycnic density centrifugation has been extensively used as a tool to isolate mucin [12,14,21,[24], [25], [26], [27], [28]] because the density of mucins is higher than the majority of other non-mucin proteins [29]. During centrifugation, a density gradient is formed and the molecules within the sample colocalize with the CsCl concentration of an equal buoyant density, separating the sample based on this property [30]. Establishing the starting density of CsCl will change the density range of the gradient, which will determine where the molecule will sediment at a given centrifugal force. Ideal starting densities and centrifugation speeds for different rotors have been identified to give an optimal final density range to isolate mucin [20,29,31]. For our workflow, we built upon a previously described method to isolate salivary MUC5B in a single ultracentrifugation step at a starting density of 1.45mg/mL ^21^. We use a SW 41-Ti swinging bucket rotor with an open top 13.2 mL tube which permits smaller sample volumes than previous protocols and allows the gradient to be fractioned by hand, eliminating the need for a fraction collector or drop counter. Consistent with sample collection and preparation, the target mucin and solubility state will determine the gradient conditions needed to optimally isolate the mucin of interest. Isopycnic isolation can be done with fixed angled rotors or using a SW 41-Ti swinging bucket rotor as discussed here.

**Fig. 1:**
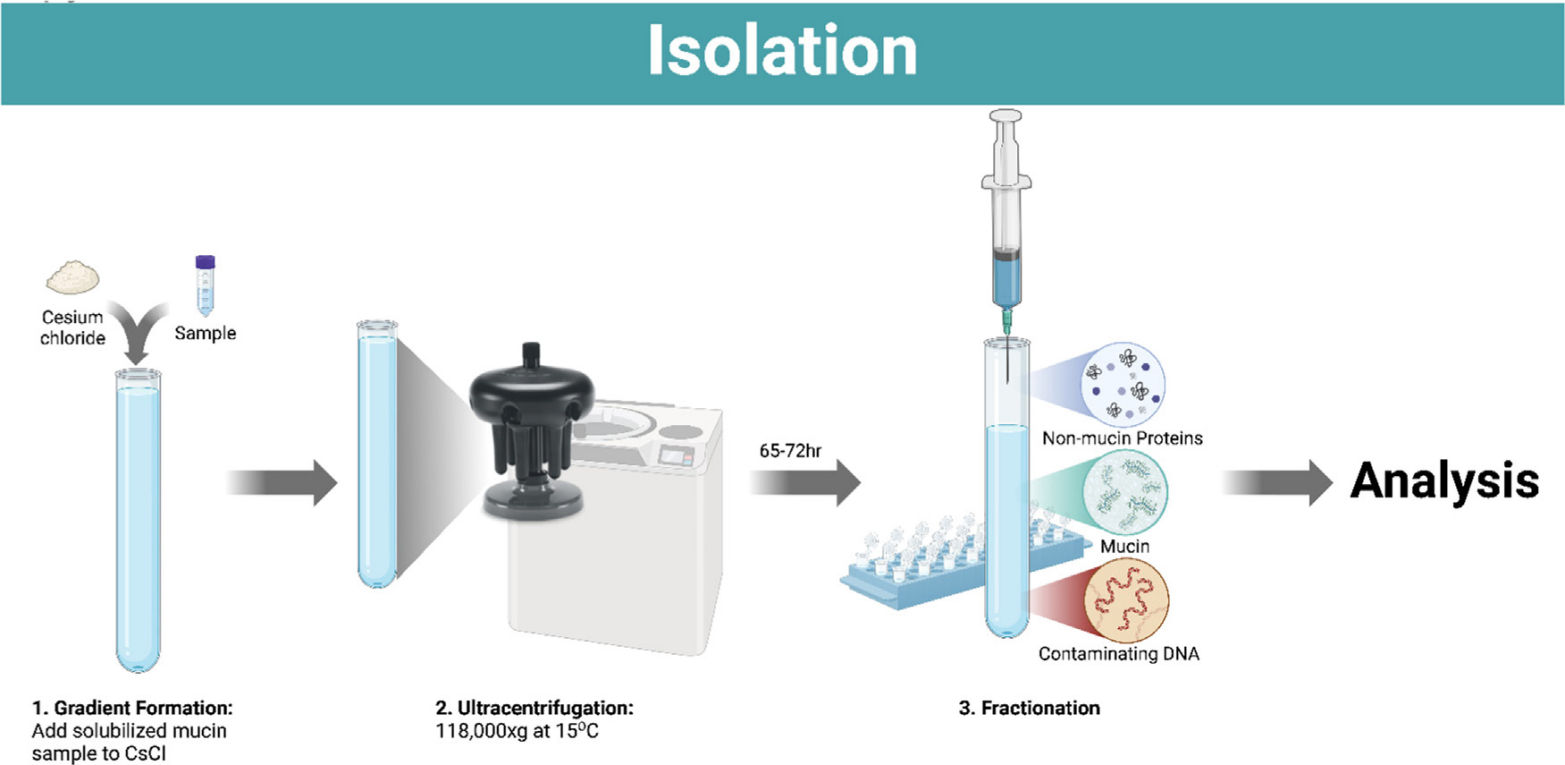
Overview of mucin isolation describing steps for gradient formation, isopycnic ultracentrifugation, and fractionation prior to analysis.

To create the isopycnic gradients, the concentration of CsCl and final volume for each gradient is needed. The desired amount of CsCl is based on the starting gradient density and calculated from the CsCl-density equation below, where 1.347 is a constant for partially purifying mucin with ultracentrifugation as determined previously [21,29]. When creating the gradient, the volume displaced by the CsCl can be determined with the below sample-volume equation, which allows you to determine the total volume of sample to add to your predetermined amount of CsCl. Fig. 1 shows the workflow of the methods that is detailed below.

CsCl−densityEquation:

CsCl(g)=v*[1.347(p)−1.347]

Sample−VolumeEquation:

$$sample\;\left(mL\right)=v-\frac{grams\;of\;CsCl}{density\;of\;CsCl}$$

$$v=final\;volume\;\left(mL\right);\;p=target\;starting\;density\;\left(\frac{mg}{mL}\right)$$

**Materials**

• Cesium Chloride (Sigma, #C3011)
• 13.2 mL Beckman Ultra-Clear Centrifuge (Beckman Coulter, #344,059)
• SW 41 Ti Swinging-Bucket Rotor (Beckman Coulter, #331,362)
• Ultracentrifuge (Beckman Coulter, Optima XPN-80)
• 1 mL Sterile Syringe (Exel, #26,048)
• 16 G Luer stub (BD, #427,561)

**Methods**

*Gradient Formation*

1. Place 1 mg of cesium chloride in 1 mL of water to determine its density.
  **Note:** Cesium chloride is hygroscopic, so its density should be checked, regularly.
2. Determine amount of CsCl needed: The open top 13.2 mL Beckman Ultra-Clear Centrifuge tube will hold a final volume of 12mL. For one 12 mL gradient at 1.45mg/mL, 7.27*g* of CsCl will be used.
  **Note:** For alternative volume, density, or other gradient conditions, the CsCl-density equation can be used to calculate the gram amount of CsCl needed.
3. Determine amount of CsCl that will displace volume: Assuming the approximate density of CsCl, 3.99mg/mL, and the 12 mL final volume, 1.82 mL of the 12 mL will be displaced by the CsCl – thus only 10.18 mL of sample will need to be added to 7.27*g* of CsCl for one 12 mL gradient at 1.45mg/mL.
  **Note:** The volume displaced by the CsCl can be determined with the sample-volume equation.
4. Add solubilized sample (*Sample Collection and Preparation*, Step 6) to CsCl and mix gently at room temperature until all of the salt is solubilized.
5. Distribute 12 mL of gradient solution into ultracentrifuge tube(s).

*Ultracentrifugation*

1. Place gradient(s) in SW 41-Ti swinging bucket rotor and into a 15 °C ultracentrifuge.
2. Set ultracentrifuge to 118,000x*g* for 65- to 72-hours.
3. Remove rotor and transport ultracentrifuged tubes into 4 °C.
  **Note:** It is important to not jar the rotor or the ultracentrifuged tubes prior to fractionation. Immediate fractionation following ultracentrifugation is recommended and to not let sit for prolonged periods of time.

*Fractionation*

1. Prepare a 1 mL syringe with a 16 G Luer stub to manually fractionate the gradient.
2. Ensuring the tip of the Luer stub is just below the meniscus of the gradient, draw up 500 µL fractions.
  **Note:** If multiple gradients were run, utilize a new syringe and Luer stub per gradient.
3. Repeat steps 2–3 for all remaining fractions.
4. Store fractions in 4 °C and reserve for fraction analysis.

#### Analysis

Following fractionation, individual fraction analysis is necessary to validate the gradient distribution along with assessment of mucin- and non-mucin-containing fractions. To validate the gradient, the density of each fraction is determined with a microbalance and returned to the original fraction collection, minimizing waste of sample. We used a NanoDrop spectrophotometer to assess the non-mucin containing fractions from the mucin sample at an absorption of 280nm*. We use MUC5B immunoblotting to visualize mucin from our salivary sample across the gradient. As an alternative method that is similarly accurate, dot blotting on nitrocellulose followed with Periodic-Acid Schiff's (PAS) staining can also be used (see Method Validation Results). PAS stains carbohydrates a magenta color and is often used to visualize mucins [28,32]. Once fractions are assessed, the fractionated sample can be pooled together and buffer exchanged into a Storage Buffer (10mM Tris, 10 mM NaCl; pH 7.4).

*280 nm will recognize aromatic amino acids commonly found in proteins and aromatic nucleotide rings in DNA. Mucins exhibit minimal absorbance at 280 nm, and this technique has been used previously to assess non-mucin molecules in these mucin isolation processes[28].

**Materials**

• Micro Balance (Cole-Parmer, Mettler Toledo XP6 Excellence Plus XP Micro Balance)
• 0.2 mL Tubes (ThermoFisher Scientific, #AB0620)
• Bio-Dot SF Apparatus (Bio-Rad, #1,706,542)
• 0.45µm Nitrocellulose Blotting Membrane (Bio-Rad's, #1,620,115)
• Denaturing buffer – pH 7.4
  • 4 M Guanidine-HCl (Sigma, #63,272)
  • 0.1 M Trizma Base (Sigma, #RDD008)
  • 25 mM Dithiothreitol (DTT) (Sigma-Aldrich, #43,816)
• 10X Tris-Buffered Saline (TBS) – pH 7.6
  • 200 mM Trizma Base (Sigma, #RDD008)
  • 1.5 M Sodium chloride (Fischer, #L-23,071)
• TBS-T
  • 1 % Tween-20 (Fisher, #AAJ20605AP)
  • 1X TBS
• Blocking Buffer
  • 5 % Milk (SANALAC Nonfat Dry Milk)
  • 1 % Tween-20 (Fisher, #AAJ20605AP)
  • 1X TBS
• MUC5B Primary Antibody (NovusBio, #4A10-H2)
• IRDye 680RD Goat anti-Mouse IgG (LicorBio, #926–68,070)
• Periodic Acid Solution
  • 1 % (v/v) Periodic acid (Sigma-Aldrich, #P7875)
  • 3 % (v/v) Acetic acid (Fisher, #A35–500)
• PAS Wash
  • 0.1 % (w/v) Sodium metabisulfite (Sigma-Aldrich, #255,556)
  • 10 mM Hydrochloric Acid (Fisher, #SA56–500)
• Schiff's Reagent (Sigma, #3,952,016)
• LiCor Odyssey M Imaging System
• 10 kDa filters (Millipore-Sigma, #UFC501008)
• Eppendorf Centrifuge 5425 R (Millipore-Sigma, #EP5406000445)
• Storage buffer – pH 7.4
  • 10 mM Trizma Base (Sigma, #RDD008)
  • 10 mM Sodium Chloride (Fischer, #L-23,071)
• Optional: Sodium azide (Sigma-Aldrich, #S2002)
• Optional: 1X Protease inhibitors
  • EDTA-free Protease Inhibitor Cocktail (Millipore-Sigma, #11,836,170,001)
  • Protease Inhibitor Cocktail (Millipore-Sigma, #11,836,153,001)

**Methods**

*Gradient Validation*

1. Pre-weigh 0.2 mL tubes on a micro balance and add 200 µL of each sample fraction to the pre-weighed tube.
2. Allow samples to reach room temperature prior to weighing.
  **Note:** Return samples to 4 °C when not in use.
3. Weigh on micro balance to find weight on the sample volume.
4. Record weight and return sample to original fraction. Repeat for all fractions.
5. Scale each weight by five to get mg/mL units and plot against fraction number.
  **Note:** At a starting density of 1.45mg/mL CsCl, fractions are expected to center around this density, with fractions 1 to 24 to show consistently, increasing densities.

#### Mucin probing

*Slot-blotting*

1. Soak the 0.45µm nitrocellulose blotting membrane in TBS for 10-minutes prior to building the slot-blotting apparatus.
2. Create 1X TBS.
3. Load 100 µL TBS into each slot and place under a vacuum until all wells are dry.
4. Prepare fraction samples by diluting 6 µL of sample in Denaturing Buffer to a final volume of 200 µL and load into each slot of the slot-blotting apparatus.
  **Note:** If lower volumes of saliva were collected prior to ultracentrifugation, a more concentrated dilution may be needed.
  **Note:** DTT should be added fresh to denaturing buffer.
  **Note:** Any unused wells should be filled with TBS, to avoid excessive drying out of the blotting paper.
5. Place under a vacuum until all wells are empty.
6. Add 200 µL of TBS to each well, and place under vacuum until all wells are empty.
7. Remove membrane and let sit in TBS for 5-minutes.
  **Note:** This will aid in removing any residual Denaturing Buffer
8. Proceed to probing.

*Dot-blotting*

1. Pipette 10 µL aliquots of undiluted sample onto 0.45µm nitrocellulose blotting membrane, ensuring approximately 1-inch of space between each dot for the sample to spread.
2. Leave paper to air-dry until complete evaporation of the sample.
3. Wet the membrane in distilled water for 5-minutes.
4. Proceed to probing.

*Probing – Immunoblotting*

1. Place blot in Blocking Buffer for 30-minutes.
2. Transfer membrane into MUC5B antibody in Blocking Buffer (1:500) at 4 °C overnight.
3. Wash thrice with TBS-T at 5-minute increments.
4. Incubate with Goat anti-Mouse IgG IRDye-680 in Blocking Buffer (1:10,000) for 1-hour.
5. Wash thrice with TBS-T at 5-minute increments.
6. Image with LiCor Odyssey at 700 nm channel.

*Probing – Periodic-Acid Schiff's (PAS) Staining*

1. Incubate 0.45µm nitrocellulose blot paper in Periodic Acid Solution for 30-minutes.
2. Wash in distilled water twice in 5-minute increments.
3. Wash membrane in PAS Wash solution twice in 5-minute increments.
4. Incubate the membrane in Schiff's reagent for 15-minutes.
5. Wash membrane in PAS Wash solution twice in 5-minute increments.
6. Leave blot to air-dry and image with LiCor Odyssey at 525 nm Epi channel or using any colorimetric imager.

*Pooling & Storage*

1. Using fraction assessments, pool fractions as desired.
2. Load sample into a 10 kDa conical filter(s).
3. Buffer exchange from the CsCl into the Storage Buffer using the 10 kDa conical filters.
4. Bring volume up to desired final concentration.
  **Note:** For the reported MUC5B isolation, a final concentration of 200µg/mL is made.
  **Note:** For long-term storage, −80 °C storage is recommended. For medium- to short-term storage at 4 °C or −20 °C, addition of 0.02 % NaN_3_ and protease inhibitors are recommended.

## Quantitation of mucin using a bovine submaxillary mucin standard

#### Mucin Bradford

Once a mucin sample is collected, quantitation is needed prior to further sample evaluation. In our studies, we found traditional BSA-based protein quantitation to underestimate the mucin amount due to the high glycan content of the mucin sample (see Method Validation Results). Moreover, a decreased abundance of basic amino acids on mucin proteins compared to BSA can affect the sample's reactivity with the Coomassie stain. The unique structure of mucin is enriched with threonine, serine, proline, and cysteine; lowering the ratio of basic residues compared the average protein. To circumvent this issue, we utilized a Bradford-based assay using bovine submaxillary mucin, which contains a similarly structured protein, to be used as a standard.

**Materials**

• Microplate Reader (BioTek Synergy MX)
• 96-well Assay Microplate (Millipore-Sigma, #CLS9017)
• Coomassie Plus Protein Assay Reagent (ThermoFisher Scientific, #1,856,210)
• Bovine Submaxillary Mucin (ThermoFisher Scientific, #J63859.ME)

**Methods**

1. Create serial dilutions of BSM standard (2.5–50µg/mL).
  **Note**: Microplate Dilutions in S. Table 1.
2. Load 150 µL of each standard dilution and a blank.
3. Create serial dilutions of sample.
4. Load 150 µL of each sample dilution.
5. Remove Coomassie Plus Reagent from fridge and allow to reach room temperature.
6. Add 150 µL of Coomassie to each well with sample/standard.
7. Triturate sample/standard with Coomassie to ensure thorough mixing.
8. Let sit covered at room temperature for 10-minutes.
9. Using a plate-reading spectrophotometer take an absorbance reading at 595nm.
10. Create a linear regression line of BSM standard concentration (µg/mL) against its corresponding absorbance to determine sample concentration based on the BSM standard curve.

## Evaluation of mucin with agarose-polyacrylamide gel electrophoresis

#### Sample preparation

**Fig. 2:**
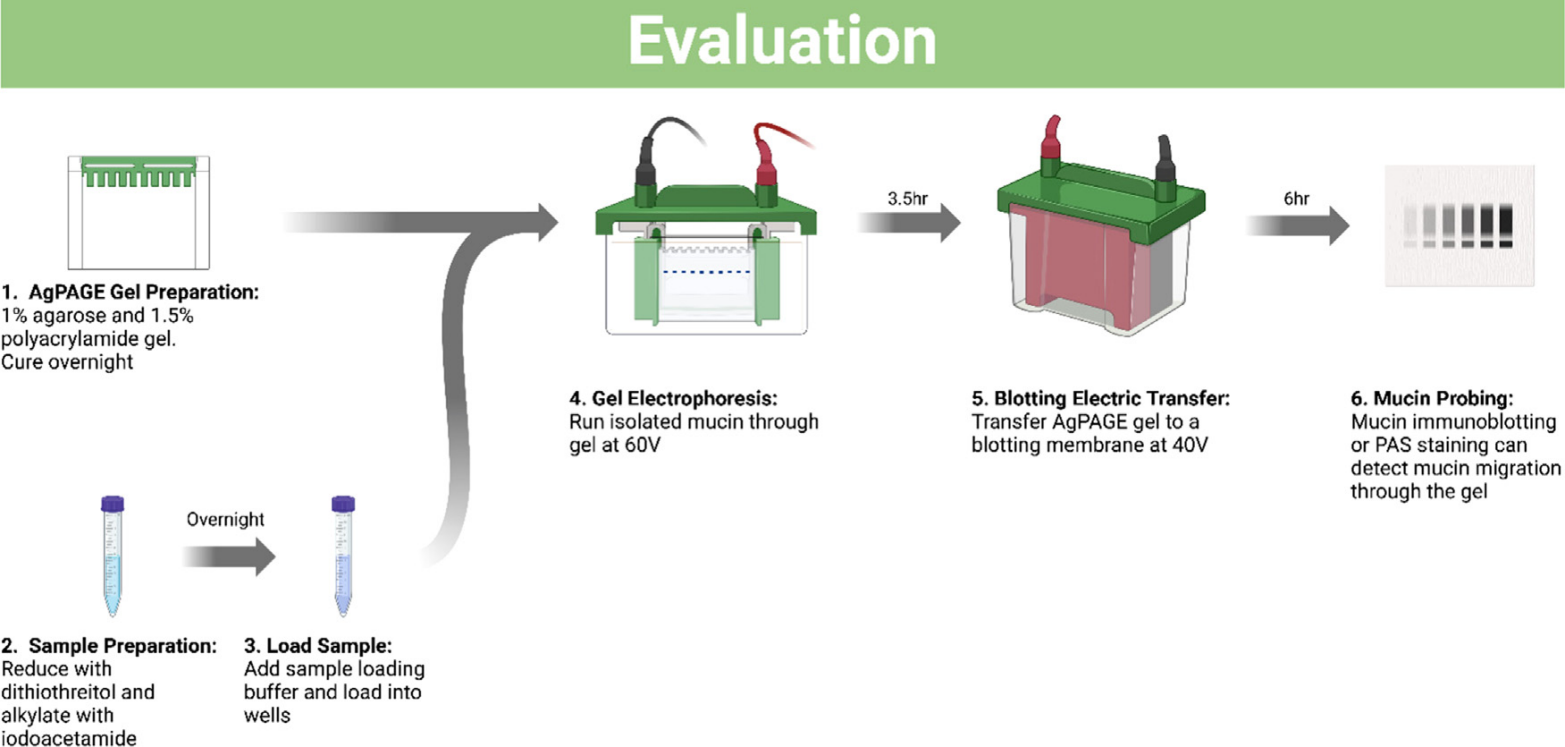
Overview of Ag-PAGE workflow with emphasis on sample and gel preparation, electrophoresis, and electric transfer prior to mucin probing for evaluation.

Protein migration through agarose-polyacrylamide gels has commonly been used to visualize mucins within a sample [[33], [34], [35]]. The use of agarose gels for mucin electrophoresis accommodates the larger size of mucins, while polyacrylamide helps increase the resolution [34]. To visualize mucin monomers and promote gel migration, mucins are reduced and alkylated prior to running on the gel. In this workflow, the migration of mucin relies primarily on its intrinsic negative charge conferred by acidic glycan residues, rather than high concentrations of anionic detergents. Similar to immunoblotting for slot-blotted membranes, PAS staining can also be used to visualize mucin migration on blotting paper as an alternative [28,32]. Fig. 2 shows the workflow of the methods that is detailed below.

**Materials**

• 10 kDa filters (Millipore-Sigma, #UFC501008)
• Eppendorf Centrifuge 5425 R (Millipore-Sigma, #EP5406000445)
• Reduction Buffer – pH 8.0
  • 6 M Urea (Fisher Scientific, #U15–500)
  • 0.1 M Trizma Base (Sigma-Aldrich, #RDD008)
  • 5 mM Ethylenediaminetetraacetic Acid (EDTA) (Sigma-Aldrich, #SLCC7167)
  • 10 mM Dithiothreitol (DTT) (Sigma-Aldrich, #43,816)
  • 20 mM Iodoacetamide (ThermoFisher Scientific, #122,270,050)
• Isotemp Water Bath (Fisher Scientific, GPD10)
• Sample Loading Buffer – pH 8.0
  • 400 mM Trizma Base (Sigma-Aldrich, #RDD008)
  • 10 mM Ethylenediaminetetraacetic Acid (EDTA) (Sigma-Aldrich, #SLCC7167)
  • 1 % Sodium Dodecyl Sulfate (SDS) (Fisher Scientific, #BP-166–500)
  • 50 % Glycerol (Invitrogen, #15,514–011)
  • 0.01 % Bromophenol blue (Fischer Biotech #BP114–25)

**Methods**

1. Load sample into a 10 kDa conical filter(s).
2. Buffer exchange from the Storage Buffer into a Reduction Buffer using 10 kDa conical filters.
3. Bring volume up to a desired concentration for loading.
  **Note:** For the reported MUC5B isolation, a final concentration of 400µg/mL is made.
4. Add 10 mM DTT to the sample and place in a warm water bath at 37 °C for 5-hours.
5. Remove from water bath and add 20 mM iodoacetamide.
6. Let sit covered at room temperature overnight.
7. Add 10X Sample Loading Buffer to the sample.
8. Let sit for 10-minutes prior to loading onto gels.

#### Ag-PAGE Preparation, Electrophoresis, and Transfer

**Materials**

• Mini-PROTEAN Tetra Cell Casting Module (1.5 mm) (Bio-Rad, #1,658,021)
• 25 mL Disposable Serological Pipet (Fisher Scientific, #13–678–11)
• 1.5X Gel Buffer – pH 8.1
  • 6 M Urea (Fisher Scientific, #U15–500)
  • 0.5 Trizma Base (Sigma-Aldrich, #RDD008)
• Polyacrylamide Solution
  • 10 % Glycerol (Invitrogen, #15,514–011)
  • 1.5 % Acrylamide/BIS Solution (Bio-Rad, #1,610,148)
  • 1X Gel Buffer
  • 0.15 % Tetramethyl ethylenediamine (TEMED) (Bio-Rad, #161–0801)
  • 0.07 % Ammonium persulfate (APS) (Bio-Rad, #161–0700)
• Agarose Solution
  • 1 % (w/v) Agarose (SeaKem Gold Agarose, Lonza, #50,150)
  • 1X Gel Buffer
• 4-gel Vertical Electrophoresis System (Bio-Rad, #165–8001)
• PowerPac Basic Power Supply (Bio-Rad, #164–5050)
• 10X Running Buffer
  • 1.92 M Trizma Base (Sigma-Aldrich, #RDD008)
  • 10 mM Ethylenediaminetetraacetic Acid (EDTA) (Sigma-Aldrich, #SLCC7167)
  • 1 % Sodium Dodecyl Sulfate (SDS) (Fisher Scientific, #BP-166–500)
• Boric Acid (Fisher Scientific, #A73–500)
• 0.45µm Nitrocellulose Blotting Membrane (Bio-Rad, #1,620,115)
• 10x Tris/Glycine Buffer (Bio-Rad, #1,610,771)
• Methanol (Fisher Scientific, #A412P-4)
• Mini Trans-Blot Module (Bio-Rad, #1,703,935)

**Methods**

*Gel Preparation*

1. Place the assembled Mini-PROTEAN Tetra Cell Casting cast, gel combs, and glass spacers along with a 25 mL serological pipette in a 65 °C oven for 45- to 60-minutes.
2. Mix Polyacrylamide Solution, without TEMED and APS, and place in the oven to keep warm.
  **Note:** Maintaining the equipment at warm temperatures reduces the risk of instant polymerization due to abrupt changes of the mixture to cooler temperatures.
3. In a 125 mL Erlenmeyer flask, dilute gel buffer to 1X concentration and add 1 % (w/v) agarose.
4. Let the powder hydrate in solution for 20-minutes then slowly microwave until dissolved.
5. Add 0.15 % TEMED and 0.07 % APS to the warmed Polyacrylamide Solution and mix gently through inversion.
6. Combine 1 % Agarose Solution with the 1.5 % Polyacrylamide Solution and mix gently by swirling in the Erlenmeyer flask.
7. Add Agarose-polyacrylamide mixture to the heated apparatus with the warmed serological pipette, ensuring no bubbles or gaps have formed.
8. Insert combs into glass gel-casing.
9. Let gels cure at room temperature for 1-hour.
10. Allow gels to set overnight at 4 °C.
  **Note:** The gels can be used the next day or wrapped and saved for up to 2 weeks at 4 °C.

*Gel Electrophoresis*

1. Make 1X Running Buffer and pH to 8.0 with boric acid.
  **Note:** Approximately 20.5*g* of Boric Acid will be needed to pH the diluted 10X buffer. Make Running Buffer fresh for each run to ensure proper pH.
2. Arrange the Ag-PAGE gels into the 4-gel vertical electrophoresis system and add Running Buffer to the correct line.
3. Load the wells with sample.
  **Note:** For the reported MUC5B isolation, multiple dilutions of 400µg/mL of sample at a final volume of 25 µL were loaded. This concentration is based on the Mucin Bradford using BSM (Fig. 4)
4. Run electrophoresis at 60 V until dye front runs off (approximately 3.5-hours).
  **Note:** Once dye-front is off, an optional run at 80 V can be conducted for an additional 15- to 30-minutes to show greater migration within the sample.

*Blotting Electric Transfer*

1. Prepare 1X Tris/Glycine Buffer (as instructed by Bio-Rad) for transfer buffer.
2. Soak 0.45µm nitrocellulose in Tris/Glycine Buffer.
  **Note:** This method has also been conducted with the use of 0.45µm PVDF where the addition of a 10-minute activation in methanol will be needed.
3. Place gel tank in an ice bath and fill with Tris/Glycine Buffer.
4. Assemble blotting sandwich in Tris/Glycine Buffer.
5. Load sandwich into the mini trans-blot rig into a cooled, gel tank.
6. Run the transfer at 40 V for 6-hours.
7. Remove membrane, cut to size, and prepare for probing methods.

*Mucin Probing* – See above “Mucin Probing” in the Analysis Section

## Method validation

### Isopycnic isolation of mucin from saliva

**Fig. 3:**
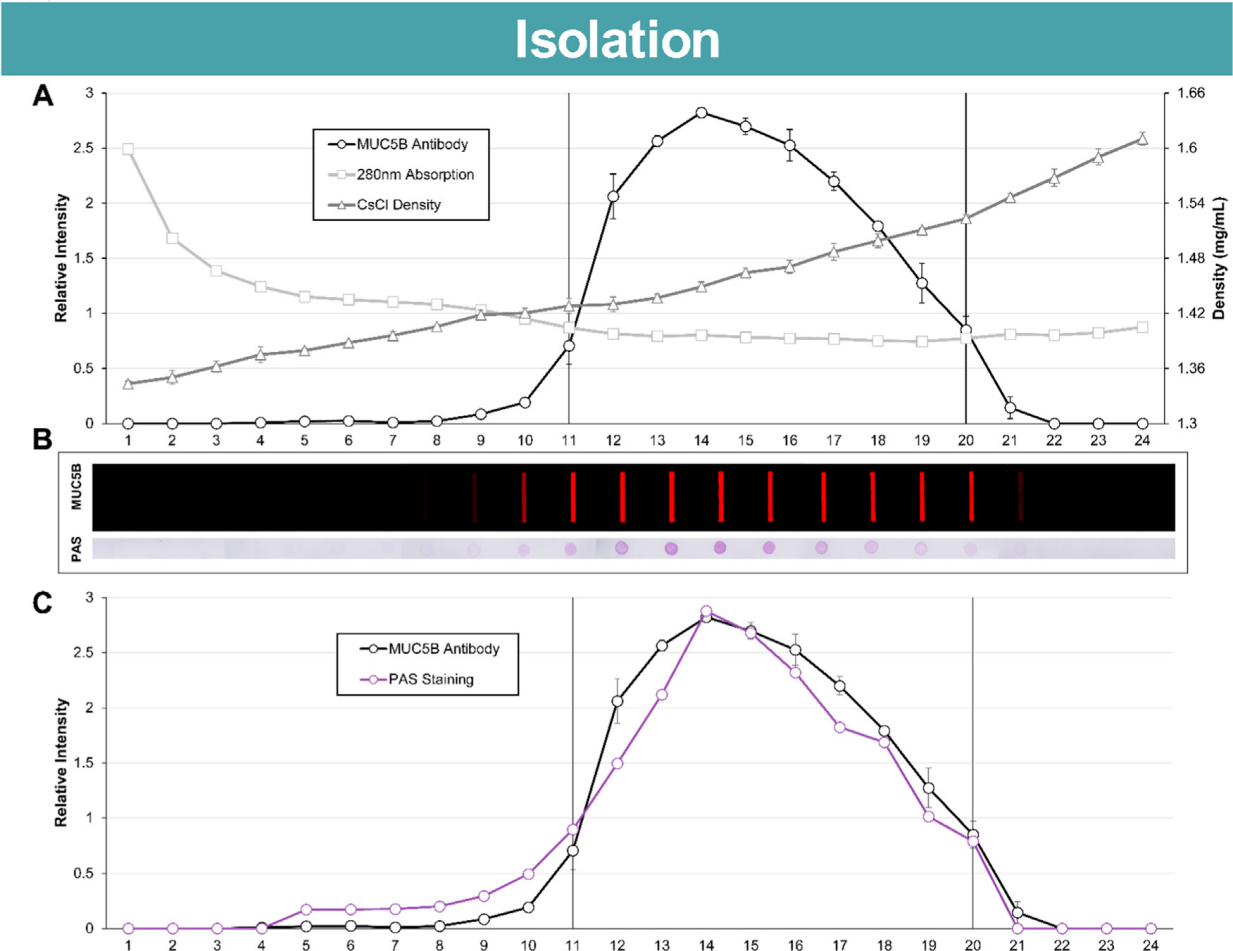
Isopycnic gradient formation, ultracentrifugation, fractionation, and fraction analysis adequately separates and identifies mucin-containing fractions. (A) Results from fraction analysis showing fractions containing MUC5B via immunoblotting (black circle), non-mucin proteins and/or DNA via 280 nm absorption (light gray square), and the increasing CsCl concentration via fraction weights (gray triangle) as an average of three gradients. Data points show mean ± SD error bars. (B) Representative slot-blot with MUC5B immunoblotting and dot-blot with PAS staining are aligned with their corresponding fraction number. (C) Averaged densitometry from the MUC5B immunoblotting method (black circle) is overlayed with the densitometry from the PAS staining method (purple circle). Dotted lines represent stringency placed on fraction collection for pooling. Images were captured on Licor Odyssey M, and the densitometry signal was calculated using Empiria Studio 3.2 Software.

**Fig. 4:**
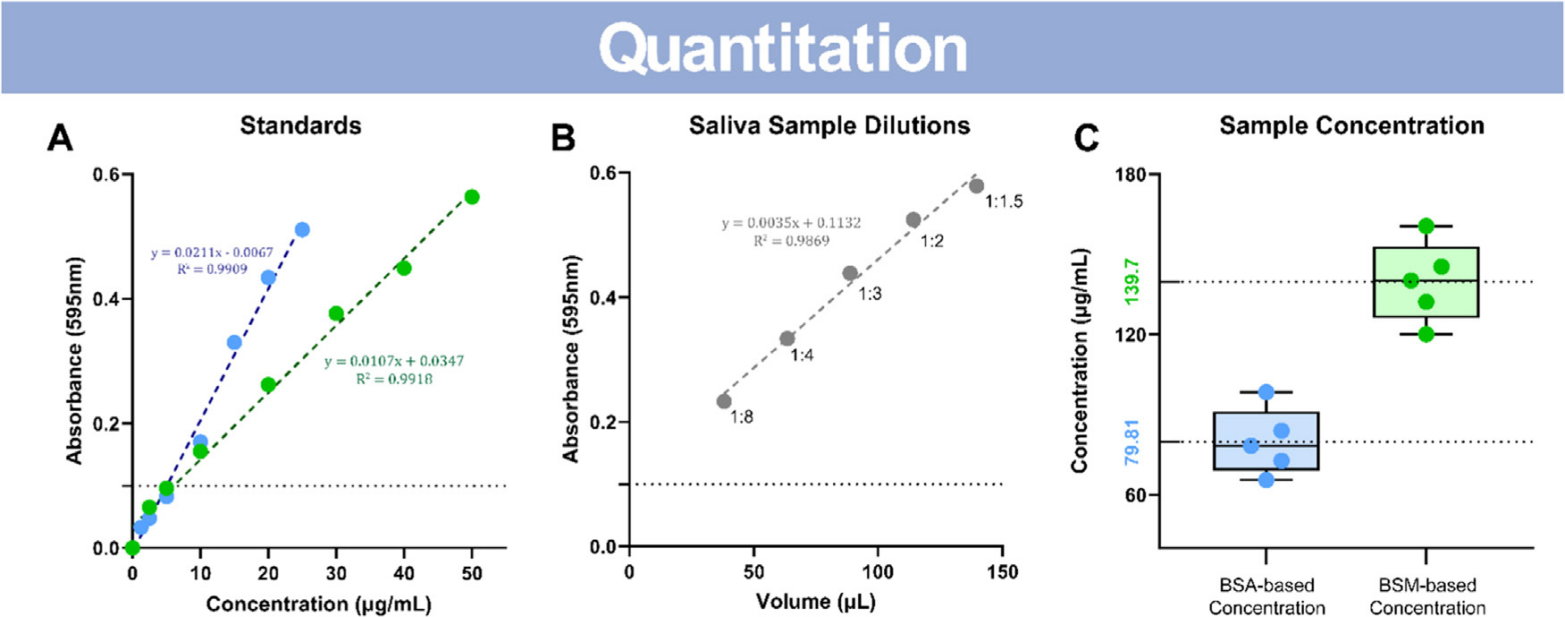
Bovine Submaxillary Mucin standard curve provides a better mucin quantitation method than commonly used Bovine Serum Albumin standard. (A) Comparison of BSA (blue) and BSM (green) standard curves and regressions across the same absorbance range. Dotted line marks absorbance value cut-off. (B) Linearity-of-dilution assessment of salivary sample at incremental volumes (µL) against their absorbance values. Dotted line marks absorbance value cut-off. (C) Box-and-Whisker plot of the calculated concentration from the BSA (blue) and BSM (green) standard for each dilution. Calculated averages are printed next to the respective dotted lines. Linear regression and statistics were performed on GraphPad Prism version 9 or greater.

Quantitation of mucin through MUC5B immunoblotting via slot-blot was overlayed on a CsCl gradient (S. Table 2). Non-mucin containing fractions were evaluated by plotting the 280 nm absorption of each fraction (S. Table 3). As shown in Fig. 3A, the isopycnic gradient, using the above methods, separated our mucin of interest, MUC5B, from the other non-mucin molecules. MUC5B immunoblotting was conducted on fractions from three separate gradients, while PAS blotting was conducted on pooled, triplicated fractions (Fig. 3B; uncropped blots in S. Fig. 1). Both PAS staining and MUC5B immunoblotting were used to demonstrate similar MUC5B sedimentation in fractions 11–20 using either detection method (Fig. 3C). These fractions can be used individually or combined for experiments. Fractions, here, were pooled and subjected to a buffer exchange in the Storage Buffer (10mM Tris, 10 mM NaCl, 0.05 % NaN_3_; pH 7.4). These pooled fractions were then subjected to protein quantification using a Mucin Bradford Assay (Fig. 4).

### Quantitation of mucin using a bovine submaxillary mucin standard

We performed a Bradford assay using BSM as a protein standard and compared it to the conventional BSA method. We chose BSM based on the structural similarities to mucin containing samples. When comparing the absorbance values of BSA and BSM over different standard concentration amounts, the dilution curves and corresponding regressions demonstrated that BSM was twice as concentrated as the BSA at comparable absorbance values (Fig. 4A; S. Table 4). Based on the absorbance values above 0.1, where the two regression lines diverge, a linearity-of-dilution assessment was made of our salivary sample. Each incremental dilution was plotted with its volume loaded and absorbance value (Fig. 4B). The absorbance values of these dilutions were then used to determine estimates of the original sample concentration using the regression model from the BSA standard and the model from the BSM standard. The mean of the estimates from the dilutions were 79.81±5.57µg/mL (SEM) for BSA and 139.7 ± 6.74µg/mL (SEM) for BSM (Fig. 4C). These data show that the mean estimates of the BSM standard was approximately 2-fold more concentrated than the BSA standard for our isolated mucin sample, suggesting that the BSM, as a more comparable protein, may be a better choice for quantitating mucin.

### Evaluation of mucin with agarose-polyacrylamide gel electrophoresis

**Fig. 5:**
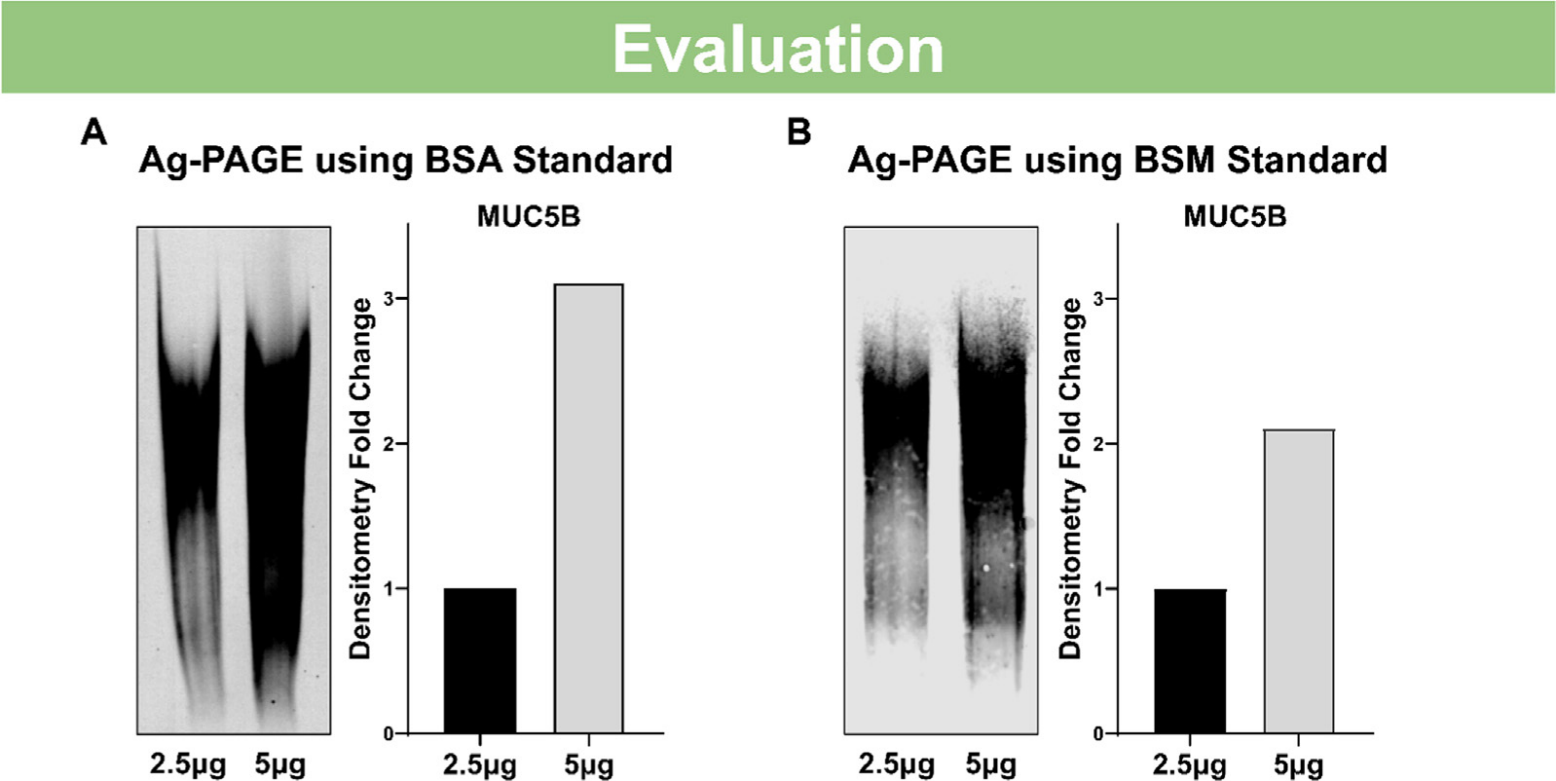
Agarose-Polyacrylamide Gel Electrophoresis in a vertical gel mold allows for mucin evaluation with common and accessible lab equipment. (A) MUC5B immunoblotting from Ag-PAGE electrophoresis and transfer of sample loaded at 2.5 µg and 5 µg determined by either the BSM standard curve with corresponding fold change of the densitometry or (B) the BSA standard curve with corresponding fold change of the densitometry. Images were captured on Licor Odyssey M, and the densitometry signal was calculated using Empiria Studio 3.2 Software and reported as fold change.

The collected, pooled, and quantitated MUC5B-enriched sample was used to validate our methods and findings. The mucin sample was loaded at either 2.5 µg and 5 µg followed by Ag-PAGE and MUC5B immunoblotting, using the above methods. The sample, quantitated by BSA prior to loading, shows more than a 3-fold change between the two different concentrations (Fig. 5A). The sample, when quantitated by BSM prior to loading, shows a more accurate 2-fold change based on the densitometry measurement compared to the difference in the two concentrations (Fig. 5B). Densitometry of the lanes highlight how the BSM quantitated sample more acurately represents the sample dilution as compared to BSA, demonstrating how the choice of standard for quantitation can affect mucin evaluation. This altogether suggests that the use of BSM as a standard can be better in quantitating mucin samples, implicating its use in further mucin studies.

## Limitations

Isopycnic ultracentrifugation for purification

It is important to note that in many cases, isopycnic ultracentrifugation does not result in complete purification of a single mucin species. Specifically, samples that contain multiple mucins with similar properties may have partially or fully overlapping sedimentation profiles and may require additional purification steps. For example, we have separated MUC5B from MUC5AC in airway secretions (data not shown). Other well-established methods using different gradient conditions have been thoroughly described to optimally isolate mucins from different families (gel-forming and tethered) that may have different solubility states under different denatured conditions. These methods can be utilized in tandem with our protocols to isolate mucins other than salivary MUC5B [20,31,33,36,37].

## Ethics statements

Salivary mucin used in these experiments was obtained from previously collected de-identified samples already approved for research, as described [35]. All experiments were performed in accordance with relevant guidelines and regulations.

## CRediT authorship contribution statement

**Hannah J. McIntire-Ray:** Conceptualization, Validation, Formal analysis, Investigation, Writing – original draft. **Elex S. Rose:** Conceptualization, Methodology, Validation, Formal analysis, Investigation, Writing – review & editing, Project administration. **Stefanie Krick:** Conceptualization, Writing – review & editing, Resources. **Jarrod W. Barnes:** Conceptualization, Methodology, Writing – review & editing, Resources, Supervision, Funding acquisition.

## Declaration of competing interest

The authors declare that they have no known competing financial interests or personal relationships that could have appeared to influence the work reported in this paper.

## Data Availability

Data will be made available on request.

## Appendix Supplementary materials

Image, application 1
