# Supplementary material for: Simple and accessible methods for quantifying isolated mucins for further evaluation
Source: MethodsX. 2025 Mar 22;14:103267. doi: 10.1016/j.mex.2025.103267 (PMC11981757; doi:10.1016/j.mex.2025.103267)
Supplement: Supplementary file 1 [file mmc1.docx]

**Supplementary material *and/or* additional information [OPTIONAL]**

| **Tube #** | **Standard Volume (µL)** | **Source of Standard** | **Diluent** | **Final Concentration (µg/mL)** |
| --- | --- | --- | --- | --- |
| **1** | **10** | **5mg/mL stock** | **990** | **50** |
| **2** | **8** | **5mg/mL stock** | **992** | **40** |
| **3** | **6** | **5mg/mL stock** | **994** | **30** |
| **4** | **500** | **Tube 2** | **500** | **20** |
| **5** | **500** | **Tube 4** | **500** | **10** |
| **6** | **500** | **Tube 5** | **500** | **5** |
| **7** | **500** | **Tube 6** | **500** | **2.5** |
| **8 (Blank)** | **--** | **--** | **500** | **0** |

## ***Supplemental Table 1:*** *Microplate Dilutions for 5mg/mL BSM*
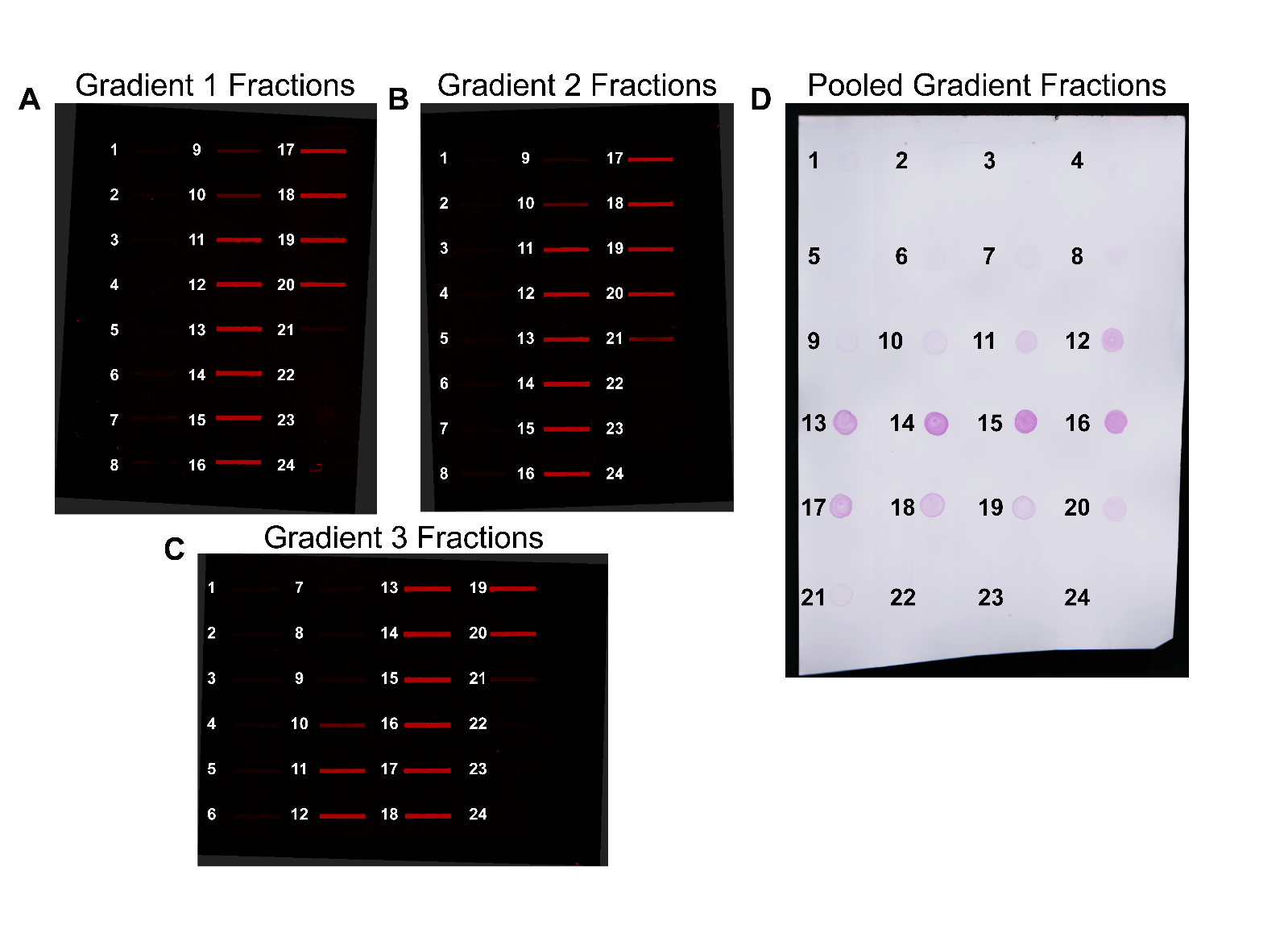


***Supplemental Figure 1:*** *Uncropped MUC5B immunoblot image of the fractions collected from the isopycnic centrifugation replicates of (A) Gradient 1, (B) Gradient 2, and (C) Gradient 3 that were slot-blotted onto 0.45µm nitrocellulose. Uncropped (D) PAS-stained blot from the three isopycnic gradients (pooled) that were dot-blotted onto 0.45µm nitrocellulose. Panels A and D were cropped and used for the representative images in Figure 3. Densitometry from these blots was used to calculate means and standard deviations in Figure 3. Blots were imaged were imaged on Licor Odyssey M at 680nm or 525nm Epi Channel, and the densitometry signal was calculated using Empiria Studio 3.2 Software.*

| **Fraction Weights – Isopycnic Ultracentrifugation** | | | |
| --- | --- | --- | --- |
| **Fraction** | **Gradient 1** | **Gradient 2** | **Gradient 3** |
| 1 | 268.482 | 268.135 | 269.400 |
| 2 | 270.328 | 268.186 | 271.676 |
| 3 | 273.784 | 270.920 | 272.643 |
| 4 | 276.135 | 272.610 | 276.306 |
| 5 | 276.415 | 275.408 | 275.903 |
| 6 | 277.796 | 277.906 | 277.131 |
| 7 | 280.429 | 278.376 | 278.734 |
| 8 | 281.276 | 280.693 | 281.417 |
| 9 | 283.760 | 282.293 | 284.935 |
| 10 | 283.925 | 282.816 | 285.489 |
| 11 | 285.208 | 283.823 | 287.780 |
| 12 | 288.041 | 285.731 | 284.088 |
| 13 | 288.220 | 286.200 | 287.665 |
| 14 | 291.238 | 289.047 | 289.075 |
| 15 | 293.558 | 291.483 | 293.521 |
| 16 | 294.693 | 292.182 | 295.478 |
| 17 | 298.244 | 294.798 | 299.122 |
| 18 | 300.515 | 297.689 | 301.255 |
| 19 | 302.622 | 301.656 | 302.260 |
| 20 | 304.998 | 303.444 | 305.479 |
| 21 | 309.722 | 308.058 | 310.042 |
| 22 | 315.186 | 310.896 | 314.522 |
| 23 | 319.527 | 315.656 | 318.966 |
| 24 | 323.763 | 320.340 | 321.947 |

## ***Supplemental Table 2:*** *Weight of 200μL aliquot of each fraction per gradient. Each weight is scaled by five to determine the density (mg/mL) of each fraction. Averages and standard deviations were used to assess gradient distribution.*

| **280nm Absorbance – Isopycnic Ultracentrifugation** | | | |
| --- | --- | --- | --- |
| **Fraction** | **Gradient 1** | **Gradient 2** | **Gradient 3** |
| 1 | 2.493 | 2.466 | 2.489 |
| 2 | 1.682 | 1.713 | 1.630 |
| 3 | 1.386 | 1.420 | 1.324 |
| 4 | 1.243 | 1.245 | 1.242 |
| 5 | 1.151 | 1.164 | 1.167 |
| 6 | 1.124 | 1.171 | 1.146 |
| 7 | 1.104 | 1.107 | 1.113 |
| 8 | 1.081 | 1.083 | 1.106 |
| 9 | 1.031 | 1.058 | 1.032 |
| 10 | 0.951 | 0.974 | 0.939 |
| 11 | 0.870 | 0.888 | 0.845 |
| 12 | 0.814 | 0.817 | 0.823 |
| 13 | 0.793 | 0.807 | 0.810 |
| 14 | 0.802 | 0.816 | 0.815 |
| 15 | 0.784 | 0.804 | 0.790 |
| 16 | 0.773 | 0.787 | 0.782 |
| 17 | 0.769 | 0.788 | 0.780 |
| 18 | 0.751 | 0.780 | 0.768 |
| 19 | 0.746 | 0.792 | 0.781 |
| 20 | 0.773 | 0.778 | 0.800 |
| 21 | 0.811 | 0.785 | 0.798 |
| 22 | 0.803 | 0.817 | 0.822 |
| 23 | 0.824 | 0.843 | 0.862 |
| 24 | 0.874 | 0.885 | 0.875 |

## ***Supplemental Table 3:*** *Absorbance values from the spectrophotometer of each fraction per gradient. These values were blank masked with milliQ water. Averages and standard deviations were used to assess non-mucin-containing fractions.*

| **Saliva Sample Dilution (Sample:Diluent)** | **Absorbance** | | **BSA Concentration (μg/mL)** | **Absorbance** | | **BSM Concentration (μg/mL)** | **Absorbance** | |
| --- | --- | --- | --- | --- | --- | --- | --- | --- |
| 1:1.5 | 0.859 | 0.862 | 25 | 0.795 | 0.794 | 50 | 0.854 | 0.847 |
| 1:2 | 0.808 | 0.805 | 20 | 0.719 | 0.716 | 40 | 0.734 | 0.739 |
| 1:3 | 0.72 | 0.721 | 15 | 0.615 | 0.612 | 30 | 0.66 | 0.667 |
| 1:4 | 0.613 | 0.619 | 10 | 0.464 | 0.444 | 20 | 0.549 | 0.55 |
| 1:8 | 0.512 | 0.518 | 5 | 0.368 | 0.365 | 10 | 0.435 | 0.45 |
| XXX | XX | XX | 2.5 | 0.331 | 0.332 | 5 | 0.377 | 0.39 |
| XXX | XX | XX | 1.25 | 0.316 | 0.318 | 2.5 | 0.345 | 0.361 |
| XXX | XX | XX | 0 | 0.285 | 0.282 | 0 | 0.282 | 0.292 |

## ***Supplemental Table 4:*** *Absorbance values in duplicate of Salivary Sample, Bovine Serum Albumin (BSA), and Bovine Submaxillary Mucin (BSM) at each dilution or concentration. Values were blank masked prior to being plotted and statistics being conducted* *on GraphPad Prism version 9 or greater.*

| **Buffer Recipes** | | | |
| --- | --- | --- | --- |
| **Solubilization Buffer** | | | *0.15M NaCl, 100mM Tris-HCl; pH 7.4** |
| Slot-Blot | | **Tris-Buffered Saline (TBS) (10X)** | *0.2M Tris-HCl, 1.5M NaCl; pH 7.6** |
|  |  | **TBS (1X)** | *20mM Tris-HCl, 150mM NaCl; pH 7.6* |
|  |  | **Denaturing Buffer** | *4M Guanidium-HCl, 0.1M Tris, 25mM DTT; pH 7.4** |
| **Storage Buffer** | | | *10mM Tris-HCl, 10mM NaCl; pH 7.4^†*^* |
| PAS  Dot-Blot | | **Periodic Acid** | *1% (v/v) Periodic acid, 3% (v/v) Acetic acid* |
|  |  | **PAS Wash** | *0.1% (w/v) Sodium metabisulfite, 10mM HCl* |
|  |  | **Schiff’s Reagent** | *Commercially Available Sigma-Aldrich – 1% (v/v) C.I. Basic Red 9 (pararosaniline), 4% sodium metabisulfite, 250mM HCl* |
| Ag-PAGE | Sample Prep | **Reducing Buffer** | *6M Urea, 0.1M Tris-HCl, 5mM EDTA; pH 8.0** |
|  |  | **Reduction** | *Reducing Buffer [6M Urea, 0.1M Tris-HCl, 5mM EDTA; pH 8.0], 10mM DTT* |
|  |  | **Alkylation** | *Reducing Buffer [6M Urea, 0.1M Tris-HCl, 5mM EDTA; pH 8.0], 10mM DTT, 20mM Iodoacetamide* |
|  | Gel Prep | **Gel Buffer (1.5X)** | *8M Urea, 2M Tris-HCl; pH 8.1** |
|  |  | **Agarose solution** | *1.5% Acrylamide/BIS, 10% glycerol, 1X Gel Buffer [0.5M Tris-HCl, 6M Urea; pH 8.0], 0.15% TEMED, 0.07% APS* |
|  |  | **Polyacrylamide solution** | *1% (w/v) Agarose, 1X Gel Buffer [0.5M Tris-HCl, 6M Urea; pH 8.0]* |
|  | Electrophoresis | **Sample Loading Buffer (10X)** | *400mM Tris-Acetate, 10mM EDTA, 1% SDS, 50% glycerol, 0.01% bromophenol blue; pH 8.0*** |
|  |  | **Running Buffer (10X)** | *1.92M Tris-HCl, 10mM EDTA, 1% SDS* |
|  |  | **Running Buffer (1X)** | *192mM Tris-HCl, 1mM EDTA, 0.1% SDS; pH 8.0**** |
|  | Transfer | **Tris-Glycine Transfer Buffer (10X)** | *Commercially Available Bio-Rad – 250mM Tris-HCl, 1.92M Glycine; pH 8.3* |
|  |  | **Tris-Glycine Transfer Buffer (1X)** | *25mM Tris-HCl, 192mM Glycine, 20% Methanol (v/v); pH 8.3* |
|  |  | **TBS-T** | *1X TBS [20mM Tris-HCl, 150mM NaCl; pH 7.6] and 1% Tween-20 (v/v)* |
| ^†^Optional Addition of 0.02% NaN_3_ and/or protease inhibitors for short-term 4°C storage  *pH buffer with Hydrochloric Acid (HCl)  **pH buffer with Acetic Acid  ***pH buffer with Boric Acid  DTT – Dithiothreitol  EDTA – Ethylenediaminetetraacetic Acid  TEMED – Tetramethyl ethylenediamine  APS – Ammonium persulfate | | | |

***Supplemental Table 5:*** *Recipes for all buffers used for methods.*
